# Evaluation of *in vitro* antileishmanial efficacy of cyclosporin A and its non-immunosuppressive derivative, dihydrocyclosporin A

**DOI:** 10.1186/s13071-020-3958-x

**Published:** 2020-02-21

**Authors:** Zhi-Wan Zheng, Jiao Li, Han Chen, Jin-Lei He, Qi-Wei Chen, Jian-Hui Zhang, Qi Zhou, Da-Li Chen, Jian-Ping Chen

**Affiliations:** 1 Department of Pathogenic Biology, West China School of Basic Medical Sciences and Forensic Medicine, Sichuan University, Chengdu, China; 2 Animal Disease Prevention and Food Safety Key Laboratory of Sichuan Province, Chengdu, China

**Keywords:** Cyclosporine A, Dihydrocyclosporin A, Cyclophilin A, Visceral leishmaniasis

## Abstract

**Background:**

New therapeutic drugs are urgently needed against visceral leishmaniasis because current drugs, such as pentavalent antimonials and miltefosine, produce severe side effects and development of resistance. Whether cyclosporine A (CsA) and its derivatives can be used as therapeutic drugs for visceral leishmaniasis has been controversial for many years.

**Methods:**

In this study, we evaluated the efficacy of CsA and its derivative, dihydrocyclosporin A (DHCsA-d), against promastigotes and intracellular amastigotes of *Leishmania donovani*. Sodium stibogluconate (SSG) was used as a positive control.

**Results:**

Our results showed that DHCsA-d was able to inhibit the proliferation of *L. donovani* promastigotes (IC_50_: 21.24 μM and 12.14 μM at 24 h and 48 h, respectively) and intracellular amastigotes (IC_50_: 5.23 μM and 4.84 μM at 24 and 48 h, respectively) *in vitro*, but CsA treatment increased the number of amastigotes in host cells. Both DHCsA-d and CsA caused several alterations in the morphology and ultrastructure of *L. donovani*, especially in the mitochondria. However, DHCsA-d showed high cytotoxicity towards cells of the mouse macrophage cell line RAW264.7, with CC50 values of 7.98 μM (24 h) and 6.65 μM (48 h). Moreover, DHCsA-d could increase IL-12, TNF-α and IFN-γ production and decrease the levels of IL-10, IL-4, NO and H_2_O_2_ in infected macrophages. On the contrary, CsA decreased IL-12, TNF-α, and IFN-γ production and increased the levels of IL-10, IL-4, NO and H_2_O_2_ in infected macrophages. The expression of *L. donovani* cyclophilin A (*Ld*CyPA) in promastigotes and intracellular amastigotes and the expression of cyclophilin A (CyPA) in RAW 264.7 cells were found to be significantly downregulated in the CsA-treated group compared to those in the untreated group. However, no significant changes in *Ld*CyPA and CyPA levels were found after DHCsA-d or SSG treatment.

**Conclusions:**

Our findings initially resolved the dispute regarding the efficacy of CsA and DHCsA-d for visceral leishmaniasis treatment. CsA showed no significant inhibitory effect on intracellular amastigotes. DHCsA-d significantly inhibited promastigotes and intracellular amastigotes, but it was highly cytotoxic. Therefore, CsA and DHCsA-d are not recommended as antileishmanial drugs.
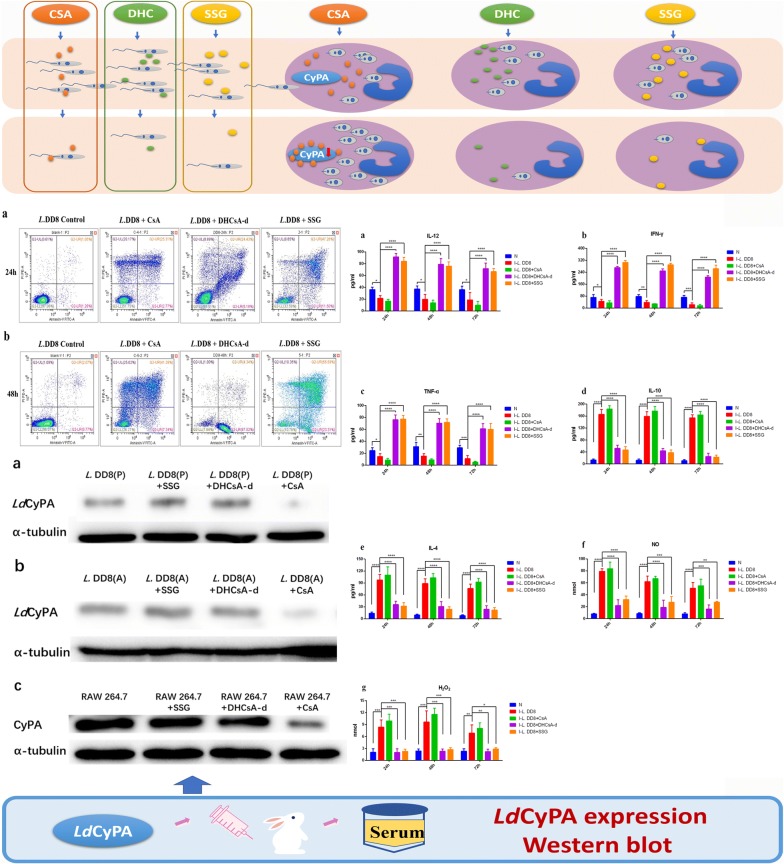

## Background

Visceral leishmaniasis (VL), a significantly neglected disease, is a systemic and chronic disease. Severe zoonosis occurs due to infections with *Leishmania donovani* or *L. infantum*. Worldwide, recurrent epidemics of VL have caused high morbidity and mortality in affected communities in 88 countries, with an estimated 400,000 new cases and 20,000 to 40,000 associated deaths [1]. VL is even now prevalent in China, in the Gansu, Sichuan, Qinghai and Shanxi Provinces and the Xinjiang Uygur Autonomous Region [2]. Pentavalent antimonials (Sb^V^) have been used as a first choice of treatment for VL in China, but currently the development of resistance against antimonials has become serious, and the severe effects of sodium stibogluconate, another anti-leishmanial drug, on the heart, liver and pancreas cannot be ignored [3]. Similar to antimonials, anti-*Leishmanial* drugs, such as amphotericin B, miltefosine and pentamidine, are also associated with severe side effects and resistance due to their long half-lives [4]. Thus, new therapeutic drugs are urgently needed.

It has always been controversial whether the immunosuppressor cyclosporin A (CsA) and its non-immunosuppressive derivatives can be used to treat VL. CsA displays anti-microbial activity against a variety of protozoan pathogens, such as *Toxoplasma* and *Trypanosoma brucei* [5, 6]. CsA has also been employed to inhibit *Leishmania* species. Previous studies have suggested that CsA has damaging effects on *Leishmania tropica* [7, 8] and *L. major* [9] *in vivo* and extracellular promastigotes of *L. tropica* were found to be sensitive to CsA [10]. Meanwhile, CsA was found to have a dose-dependent inhibitory effect on the growth of both *L. donovani* promastigotes and axenic amastigotes [11]. In addition, CsA was found to have a desired effect on VL in clinical cases [12]. It was considered to be highly efficacious in treating cytophagic histiocytic panniculitis and haemophagocytic lymphohistiocytosis, triggered by a previous visceral *Leishmania* infection, after failure of treatment with drugs, such as high-dose glucocorticoids, anakinra and etoposide [12]. However, CsA treatment was observed to convert the *L. donovani*-infected “cure” C57BL/10 mouse phenotype to a “noncure” phenotype [13]. Further, these mice exhibited a significant increase in the level of infection after 15 days of CsA-treatment [14], and the number of infected macrophages and amastigotes per macrophage in CsA-treated mice was significantly increased [15]. Based on these controversial results, we thought that CsA might inhibit the viability and proliferation of *Leishmania* promastigotes and host-free amastigotes *in vitro* and that CsA would likely play a prominent role in leishmanial infection in animals. Therefore, we hypothesized that the effect of CsA inhibition on intracellular amastigotes of *L. donovani* is counteracted by its immunosuppressive effect.

CsA exhibits its immunosuppressive action by inhibiting the production of calcineurin through binding to its intracellular specific receptor, cyclophilin A (CyPA) [16, 17]. *Leishmania donovani* can also express a variant of CyPA, known as *L. donovani* cyclophilin A (*Ld*CyPA) [18, 19], whose structural and biological functions are different from those of human CyPA. *Ld*CyPA was shown to play a pivotal role in the survival and persistence of parasites in infected tissues [20]. Thus, the inhibitory efficacy of CsA towards intracellular amastigotes can be indirectly verified by changes in the expression levels of CyPA and *Ld*CyPA.

Dihydrocyclosporin A (DHCsA-d), which is a closely related co-metabolite of CsA [21], has been reported to exert only marginal immunosuppressive activity and has been used as a control to determine the role of immunosuppression in the pharmacology of CsA-based treatment of parasitic infections [22]. Interestingly, DHCsA-d was found to also inhibit parasites *in vivo* and *in vitro* [22]. Therefore, we aimed to explore whether DHCsA-d could be used as an inhibitor of *L. donovani*.

In this study, promastigotes and intracellular amastigotes were subjected to treatment with CsA, DHCsA-d, or sodium stibogluconate (SSG) to find their effective 50% inhibitory concentrations. Next, changes in the morphology and ultrastructure of *L. donovani* promastigotes and intracellular amastigotes were assessed and cytokine and nitric oxide (NO)/hydrogen peroxide (H_2_O_2_) production by the cells was detected after CsA, DHCsA-d, or SSG treatment. Finally, the expression of *Ld*CyPA in promastigotes and intracellular amastigotes and the expression of CyPA in RAW 264.7 cells that were cultured in the presence of CsA, DHCsA-d, or SSG were detected using western blotting to verify the efficacy of DHCsA-d and CsA against *L. donovani* promastigotes, intracellular amastigotes and cells.

## Methods

### Parasite strains

*Leishmania donovani* strain MHOM/CN/IN/80/DD8 was used in this study [23]. *Leishmania* promastigotes in the logarithmic phase were cultured at 28 °C in M199 medium at pH 7.4 (Sigma-Aldrich, Saint Louis, MO, USA), supplemented with 10% fetal bovine serum (Hyclone, Logan, UT, USA) and antibiotics (Pen-Strep, 100 U/ml penicillin-100 μM streptomycin sulphate).

### Drugs

Stibogluconate sodium (MCE, Monmouth Junction, NJ, USA) was dissolved in saline to prepare a 20 mM stock. Cyclosporin A (ApexBio, Houston, TX, USA) and dihydrocyclosporin A (TRC, North York, ON, CA) were dissolved in a 20 mM stock of dimethyl sulfoxide (DMSO) (Sigma-Aldrich). For experiments, the DMSO concentration in the culture medium did not exceed 0.1%.

### *In vitro* inhibitory assays

Promastigotes, in logarithmic phase, were grown at a cell density of 1.0 × 10^6^ cells/ml, CsA, DHCsA-d, or SSG was added at different concentrations prepared from concentrated stock solutions: 5, 10, 15, 20 and 25 μM for CsA [11]; 5, 10, 15, 20 and 25 μM for DHCsA-d [22]; and 5, 10, 25, 50 and 90 μM for SSG [24]. Parasite inhibition rates were evaluated at 24 h and 48 h using flow cytometry, employing an FITC Annexin V Apoptosis Detection Kit I (BD, Franklin Lakes, NJ, USA), according to the manufacturer’s instructions. Inhibition rate (%) was calculated as follows: Inhibition rate (%) = 100% − [(No. of live parasites in treated sample/No. of live parasites in untreated control) × 100%].

To evaluate the inhibitory effects of CsA, DHCsA-d, or SSG on intracellular amastigotes, we infected macrophages of a murine macrophage stable cell line (RAW 264.7) (Jennio, Guangzhou, China) with logarithmic phase promastigotes. RAW 264.7 cells (5.0 × 10^5^ cells/per well) were plated on round glass coverslips in 24-well plates and allowed to adhere to the slides for 24 h at 37 °C, 5% CO_2_, in PRMI 1640 medium (Gibco, Franklin, TN, USA) supplemented with 10% FBS (Gibco). Adherent macrophages were infected with *L. donovani* promastigotes, at a macrophages-to-parasite ratio of 1:20 for 6 h at 37 °C, 5% CO_2_. Next, the non-infected parasites were removed by washing three times with PBS, and the infected macrophages were incubated in 37 °C in 5% CO_2_ with PRMI 1640 medium and 10% FBS without drugs for 24 h. The medium was then removed and different concentrations of CsA (3, 6, 10, 15, 20 μM), DHCsA-d (3, 6, 10, 15, 20 μM) or SSG (5, 10, 25, 50, 90 μM) dissolved in fresh medium were added, and the coverslips were incubated for 2 days. At 24 and 48 h, the glass coverslips were fixed in methanol (Solarbio, Beijing, China) and stained with Wright’s stain (Solarbio). The numbers of parasites were determined using light microscopy by counting at least 200 cells per slide. The results are expressed as means of three independent experiments. The 50% inhibitory concentration (IC_50_) was calculated for promastigotes and intracellular amastigotes by fitting the values to a non-linear curve analysis.

### Electron microscopy

CsA, DHCsA-d, or SSG-treated control promastigotes and intracellular amastigotes within the macrophages of RAW 264.7 cell line were fixed in 2.5% glutaraldehyde in 0.1 M cacodylate buffer (pH 7.2) and post-fixed in a solution containing 1% OsO_4_, 1.25% potassium ferrocyanide and 0.1 M cacodylate buffer, pH 7.2. Next, the cells were dehydrated in acetone and embedded in epoxy resin. Stained with lead citrate and observed under a Hitachi HT7700 transmission electron microscope.

### Production of polyclonal antibody of *Ld*CyPA and western blotting

Polyclonal antibodies against *Ld*CyPA were obtained to monitor protein expression. Representative peptides of *Ld*CyPA were analyzed through two predictors for B cells, BEPIPRED and BCPRED12. Among these, 8 peptides were found to be highly immunogenic. The *Ld*CyPA gene was amplified by PCR (forward primer: 5′-CGC CGA ATT CAT GTC TTA CAC GCC GCA CTA CCC CG-3′; reverse primer: 5′-CCG GTC GAC TTA AAG CTG TCC GCA GGC AGC CAC G-3′) and cloned into pET28a (Promega, Madison, WI, USA) for sequencing and expressing proteins. Polyclonal antiserum was produced in New Zealand white rabbits. The rabbits were immunized with Freund’s adjuvant (Sigma-Aldrich), containing 1500 μg of *Ld*CyPA peptide, on days 0, 15 and 30. The rabbits received three subcutaneous injections, the first in complete Freund’s adjuvant and later two in incomplete Freund’s adjuvant. Serum was obtained and antibody titers were estimated by ELISA on 25 and 42 days after the last injection.

Promastigotes, intracellular amastigotes and RAW 264.7 cells were co-cultured with the three compounds, CsA, DHCsA-d, or SSG for 48 h. Promastigotes were treated with CsA (16 μM), DHCsA-d (12 μM), or SSG (20 μM); intracellular amastigotes and RAW 264.7 cells were treated with CsA (10 μM), DHCsA-d (5 μM), or SSG (9 μM). These cells were then lysed with a protein lysis buffer supplemented with protease and phosphatase inhibitors and the protein concentration was determined by the BCA Kit (Beyotime). The extracted proteins were denatured for 5 min at 100 °C after adding appropriate amounts of loading buffer. Approximately 40 to 50 μg of proteins was loaded in each lane of a 12% polyacrylamide gel (Beyotime). Proteins were separated at 120 V until the dye front reached the bottom of the gel, followed by transfer of the gels to polyvinylidene fluoride (PVDF) membranes (EMD, MA, USA) in transfer buffer (25 mM Tris-HCl, 192 mM glycine, 20% methanol, 0.02% SDS, pH 8.3) and run at 200 mA for 60 min. The membranes were then soaked in a blocking buffer (1 × PBS, 0.1% Tween 20) and supplemented with 1% bovine serum albumin (Sigma-Aldrich) for 2 h, before incubating overnight at 4 °C individually with *Ld*CyPA polyclonal antibody, CyPA antibody (Affinity, Dublin, OH, USA) and α-tubulin antibody (Affinity) at appropriate dilutions. All the primary antibodies were used at a 1:4000 dilution and horseradish peroxidase-conjugated anti-rabbit immunoglobulin secondary antibodies (Affinity) were used at a 1:5000 dilution. The PVDF membranes were visualized with an enhanced chemiluminescence western blotting detection kit (Beyotime) and the protein bands were analyzed by densitometric studies using ImageJ software.

### Quantification of cytokines, NO and H_2_O_2_

Mouse RAW 264.7 macrophages (5.0 × 10^5^ cells/per well), adhered for 24 h at 37 °C, 5% CO_2_, in PRMI 1640 medium supplemented with 10% FBS were infected with promastigotes at a macrophages-to-parasite ratio of 1:20 for 6 h at 37 °C, 5% CO_2_, and subsequently the non-infected parasites were removed by washing three times with PBS. Infected macrophages were incubated in PRMI 1640 medium and 10% FBS at 37 °C, 5% CO_2_, without drugs for 24 h. Next, the medium was removed and CsA (10 μM), DHCsA-d (5 μM), or SSG (9 μM) (concentrations showing IC_50_ for intercellular amastigotes in this study) were added using fresh medium. The cells were further incubated for 3 days, and the medium supernatants were retrieved at 24, 48 and 72 h. Subsequently, the cytokines IL-12, IL-10, IL-4, IFN-γ and TNF-α were measured utilizing the BD OpitELA ELISA Kit (BD). NO and H_2_O_2_ concentrations in the supernatants were detected by nitric oxide and hydrogen peroxide assay kits (Beyotime), respectively.

### Cytotoxicity assessment

Cytotoxicity effects of CsA, DHCsA-d, or SSG against murine macrophages (RAW 264.7) were assessed by using the Cell Counting Kit-8 (KeyGEN, Shanghai, China). Murine macrophages were cultivated in a 96-well plate using PRIM 1640 medium containing 10% FBS and maintained at 37 °C, 5% CO_2_, for 24 h. Next, different concentrations of the drugs (7, 10, 20, 50 and 100 μM for CsA and DHCsA-d; 60, 75, 90, 105 and 120 μM for SSG) were added. The cytotoxicity was measured at 24 and 48 h following a standard protocol.

### Statistics

All experiments were carried out in triplicates and repeated at least three times and the figures shown are representative of these experiments. IC_50_ were calculated according to a nonlinear regression using a log inhibitor-*versus* response equation with 95% confidence intervals in the GraphPad Prism 6.0 software. The data were analyzed using Student’s t-test for comparison of two groups and data values were expressed as the mean ± standard deviation (SD). Significant differences were determined and designated with asterisks as follows: *P < 0.05; **P < 0.01; ***P < 0.001 and ****P < 0.0001.

## Results

### Susceptibility testing

**Fig. 1:**
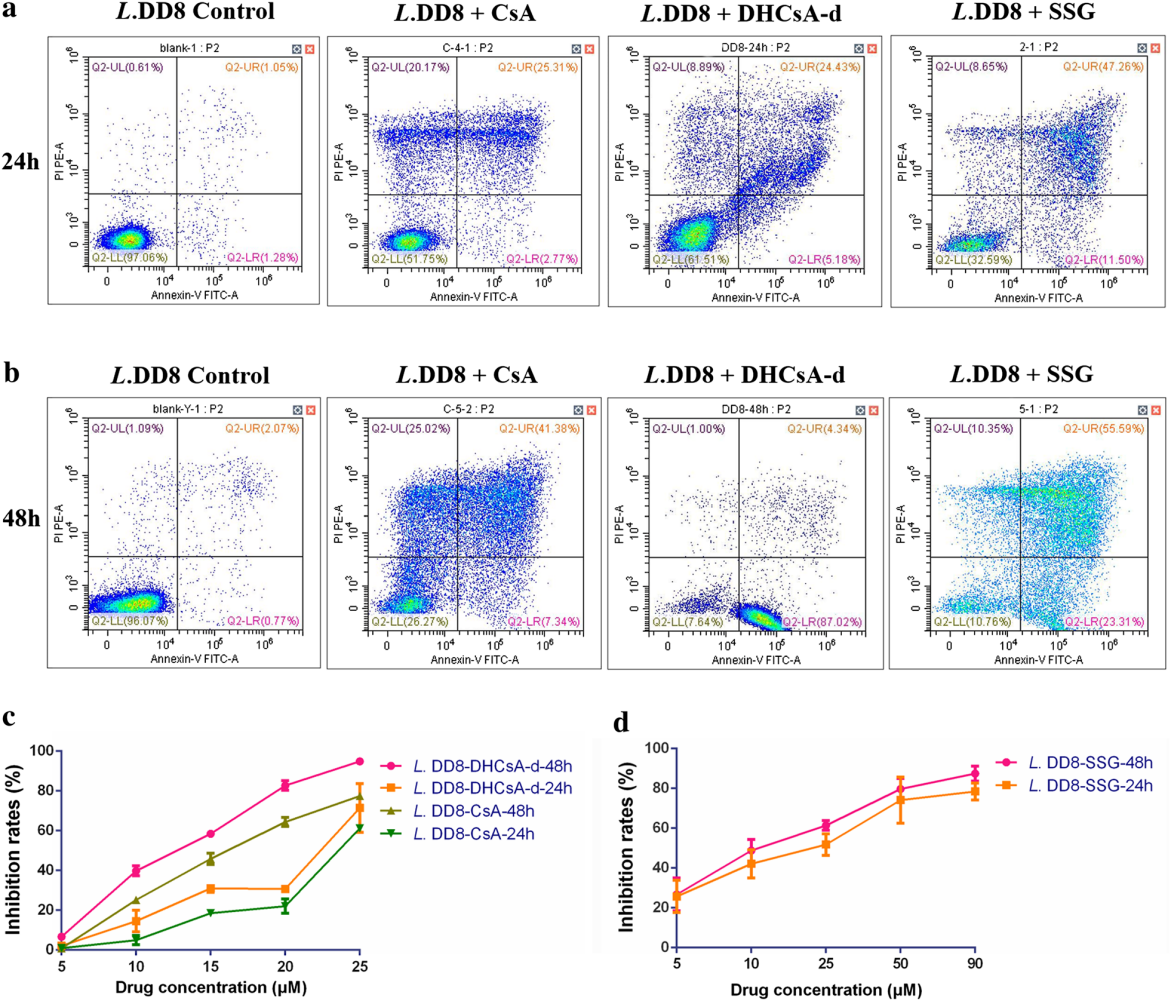
CsA, DHCsA-d, or SSG treatment affects promastigotes multiplication and inhibition rates of promastigotes. Live promastigotes (LL quadrant) were assayed by flow cytometry at 24 h (**a**) or 48 h (**b**) after treatment with the individual drugs. **c** Inhibition rates of promastigotes treated with different concentration of CsA and DHCsA-d at 24 h and 48 h. **d** Inhibition rates of promastigotes treated with different concentration of SSG at 24 h and 48 h

**Fig. 2:**
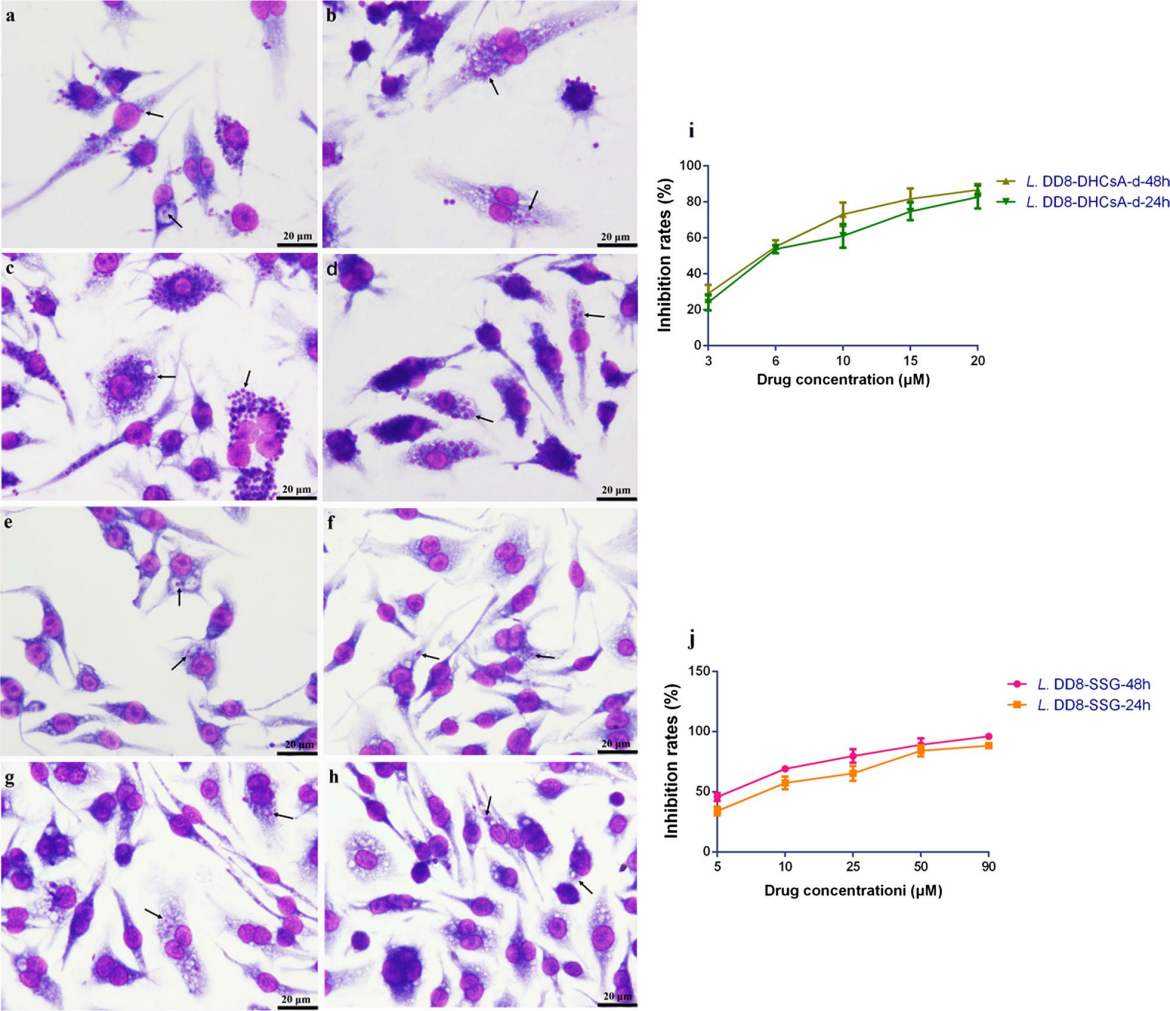
Wright’s staining of intracellular amastigotes in cells after treated with three drugs and inhibition rates of intracellular amastigotes. Untreated control amastigotes after 24 h (**a**) and 48 h (**b**) of infection. Amastigotes treated with CsA (10 μM) for 24 h (**c**) and 48 h (**d**). Amastigotes treated with DHCsA-d (5 μM) for 24 h (**e**) and 48 h (**f**). Amastigotes treated with SSG (9 μM) for 24 h (**g**) and 48 h (**h**). Inhibition rates of intracellular amastigotes treated with different concentrations of DHCsA-d (**i**) or SSG (**j**) at 24 h and 48 h. The arrows indicate amastigotes

We treated promastigotes with different concentrations of CsA, DHCsA-d, or SSG for 24 and 48 h; this was followed by flow cytometry using annexin V-FITC and PI labeling. All the three drugs could inhibit promastigotes at 24 h (Fig. 1a) and their inhibitory effect was enhanced over a period of time (Fig. 1b). The inhibition rates were calculated (Fig. 1c, d). Intracellular amastigotes were treated with different concentrations of CsA, DHCsA-d, or SSG for 24 and 48 h followed by microscopy to calculate inhibition rates (Fig. 2i, j). The results of Wright’s staining suggested that compared to the numbers of untreated amastigotes (Fig. 2a, b), the numbers of DHCsA-d-treated or SSG-treated intracellular amastigotes were reduced after 24 h of treatment (Fig. 2e, g) and were basically absent after 48 h of treatment (Fig. 2f, h), leaving several different sizes of parasitophorous vacuoles in the cells. Interestingly, on the contrary, the number of intracellular amastigotes was significantly increased in CsA-treated cells after a 24 h (Fig. 2c) and 48 h (Fig. 2d) treatment compared with that in the control group cells.

Based on the inhibitory effect of different concentrations of the drugs assessed, IC_50_ values of these three drugs were calculated (Table 1). The IC_50_ values of DHCsA-d-treated promastigotes were 21.24 μM (24 h) and 12.14 μM (48 h). Likewise, DHCsA-d had a striking inhibitory effect on the growth of intercellular amastigotes. The IC_50_ values of DHCsA-d-treated amastigotes were 5.23 μM and 4.84 μM at 24 and 48 h, respectively. CsA was also active against promastigotes, showing an IC_50_ value of 23.44 μM at 24 h and 15.88 μM at 48 h. The IC_50_ value of CsA towards intercellular amastigotes was not determined because we found that the number of amastigotes in the CsA-treated group was increased compared with that in the control group. The SSG group showed IC_50_ values of 32.33 μM (24 h) and 20.23 μM (48 h) for promastigotes and 9.51 μM (24 h) and 8.27 μM (48 h) for amastigotes.

**Table 1:** IC_50_ of CsA, DHCsA-d, or SSG used for treatment of leishmanial promastigotes and intracellular amastigotes

Compound	Promastigotes IC_50_		Amastigotes IC_50_	
	24 h	48 h	24 h	48 h
	Mean ± SD (µM)	Mean ± SD (µM)	Mean ± SD (µM)	Mean ± SD (µM)
CsA	23.44 ± 0.3	15.88 ± 0.4	nd	nd
DHCsA-d	21.24 ± 1.2	12.14 ± 1.1	5.23 ± 2.1	4.84 ± 0.4
SSG	32.33 ± 1.6	20.23 ± 1.4	9.51 ± 0.5	8.27 ± 0.1

*Note*: Data are represented as the mean ± SD of three independent experiments

*Abbreviations*: IC_50_, 50% inhibitory concentration; nd, not determined; SD, standard deviation

### Drugs altered mitochondrial ultrastructure

**Fig. 3:**
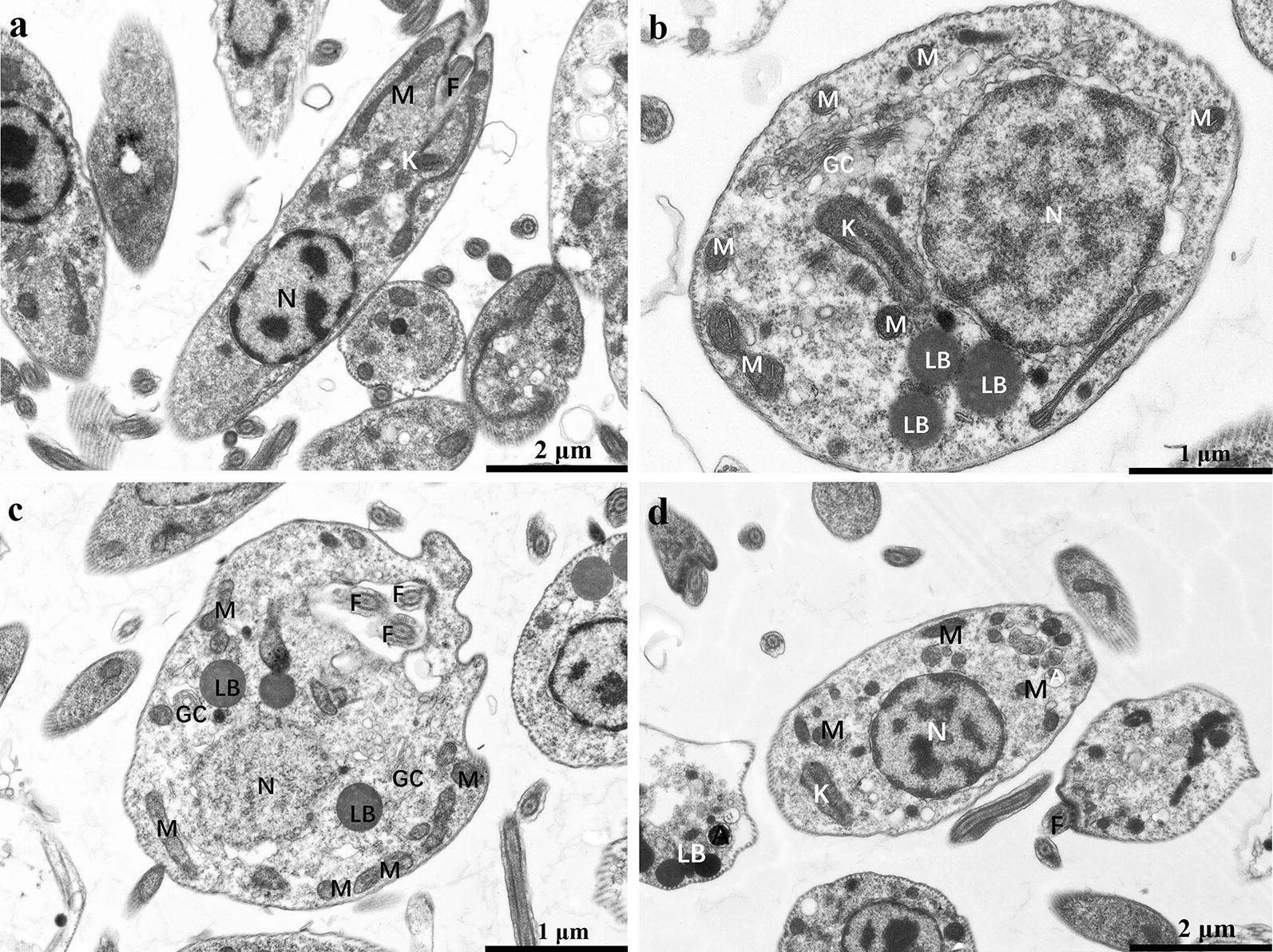
Ultrathin sections of promastigotes. Untreated control promastigotes (**a**) and promastigotes treated with CsA (16 μM) (**b**), DHCsA-d (12 μM) (**c**), or SSG (20 μM) (**d**) for 48 h. There were several alterations observed, such as changes in the shape of the promastigotes and mitochondria (**b**–**d**): the promastigotes became rounded and mitochondria became rounded and swollen and several lipid bodies were present (**b**, **c**) and multiple flagellum (**c**). *Abbreviations*: F, flagellum; GC, Golgi complex; K, kinetoplast; LB, lipid bodies; M, mitochondrion; N, nucleus

CsA, DHCsA-d, or SSG induced significant morphological changes in promastigotes, including increased aggregate formation, oval cell shape and shortened and motionless flagellae (Fig. 3b–d). The ultrastructure of the main organelles of *L. donovani* was also significantly altered after treatment with CsA, DHCsA-d, or SSG, especially that of the mitochondria, which appeared completely disorganized (Fig. 3b–d). As shown in Fig. 3a, the control promastigotes presented normal ultrastructure for mitochondria and nuclei. Moreover, there was an abnormal increase in the number of flagellae in DHCsA-d-treated promastigotes (Fig. 3c).

**Fig. 4:**
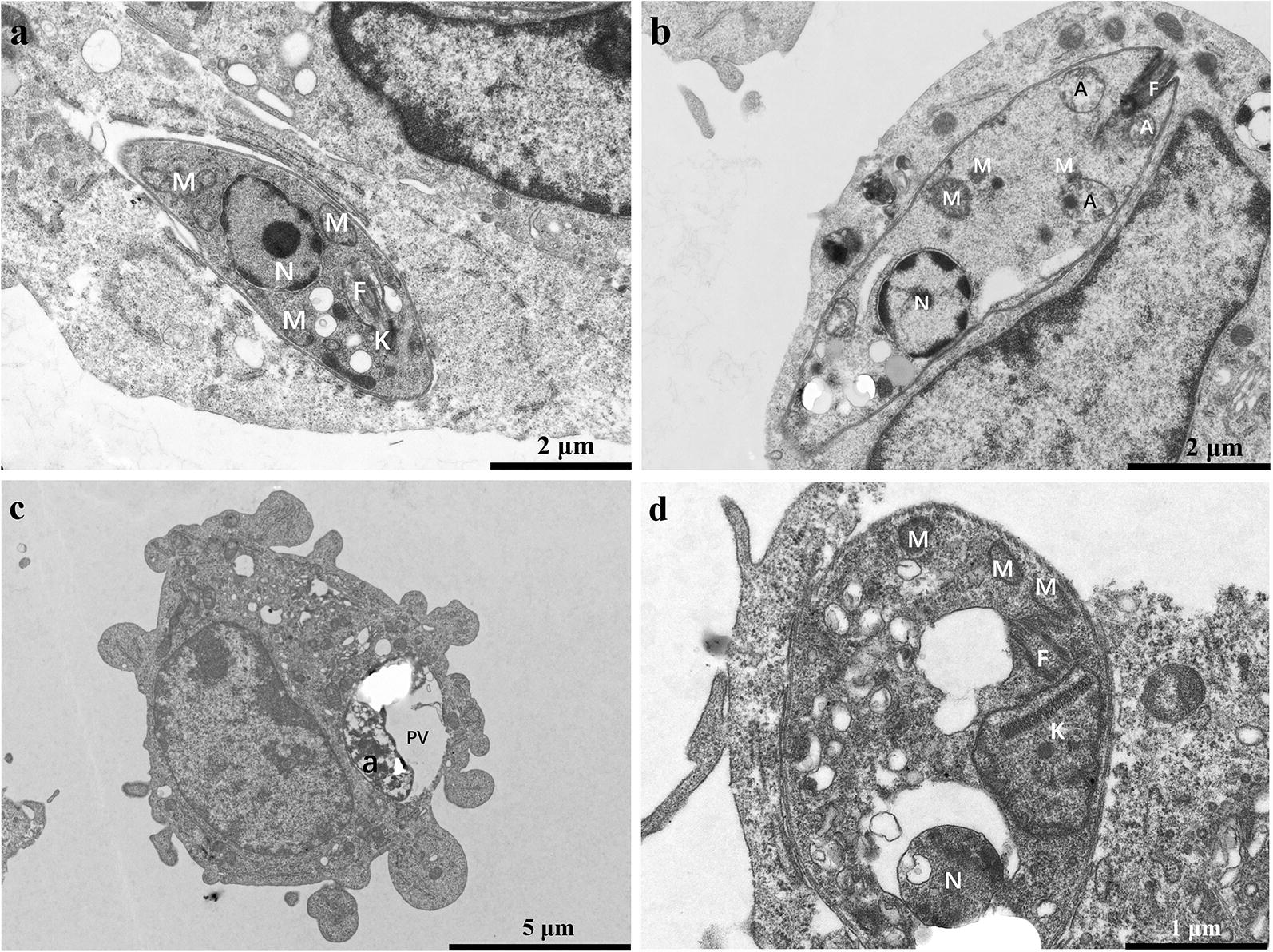
Ultrathin sections of amastigotes. Untreated control amastigote (**a**) and amastigotes treated with CsA (10 μM) (**b**), DHCsA-d (5 μM) (**c**), or SSG (9 μM) (**d**) for 48 h. Several different ultrastructural alterations were observed, such as autophagosomes (**b**) in the cytoplasm; intense disorganization and swelling can be observed in nuclei of the parasites (**d**), degraded amastigotes in large parasitophorous vacuoles were observed (**c**). *Abbreviations*: a, amastigote; A, autophagosome; F, flagellum; K, kineplast; M, mitochondrion; N, nucleus; PV, parasitophorous vacuole

The untreated intracellular amastigotes presented normal ultrastructure for nuclei and mitochondria (Fig. 4a). The mitochondria of intracellular amastigotes treated with DHCsA-d or SSG appeared disintegrated (Fig. 4c, d). Almost half of the DHCsA-d-treated intracellular amastigotes were degraded in parasitophorous vacuoles (Fig. 4c). CsA-treated intracellular amastigotes remained unchanged, unlike the CsA-treated promastigotes, which did not show significant changes as compared to the untreated group (Fig. 4b).

### The effect of CsA, DHCsA-d, or SSG on cytokine, NO and H_2_O_2_ production

**Fig. 5:**
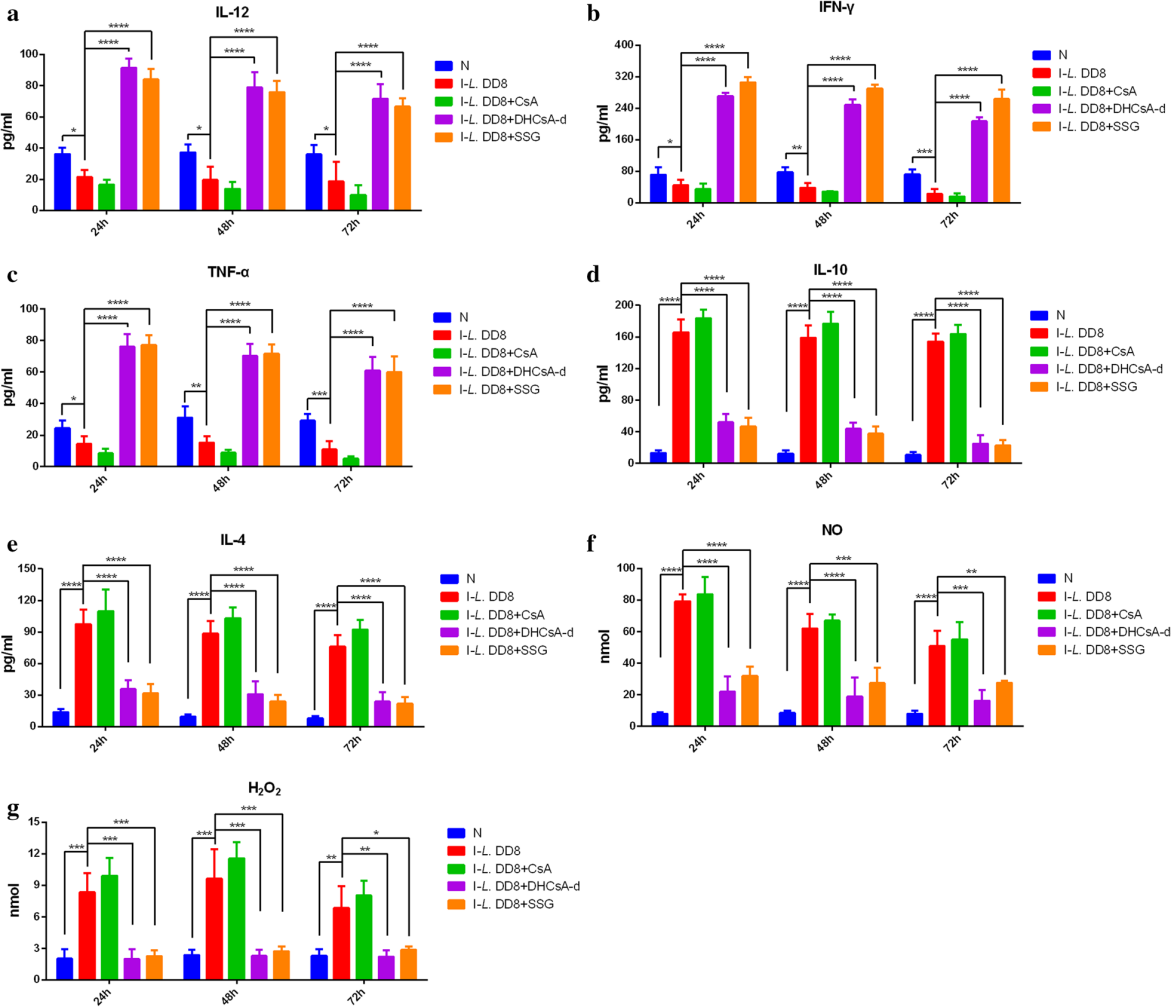
Estimation of cytokines, NO and H_2_O_2_ in culture supernatants of RAW 264.7 by ELISA. Levels of interleukin 12 (IL-12) (**a**), interferon γ (IFN-γ) (**b**), tumor necrosis factor α (TNF-α) (**c**), interleukin 10 (IL-10) (**d**), interleukin 4 (IL-4) (**e**), nitric oxide (NO) (**f**) and hydrogen peroxide (H_2_O_2_) (**g**) production in culture supernatants was measured. *Abbreviations*: N, non-infected macrophages; I-*L*. DD8, macrophages infected with *L*. DD8; I-*L*. DD8 + SSG, SSG adding after macrophages infected with *L*. DD8; I-*L*. DD8 + CsA, CsA adding after macrophages infected with *L*. DD8; I-*L*. DD8 + DHCsA-d, DHCsA-d adding after macrophages infected with *L*. DD8. Data are the mean ± standard error of the mean and represent the values of one of the 3 independent experiments. *P < 0.05, **P < 0.01, ***P < 0.001, ****P < 0.0001

CsA, DHCsA-d, or SSG were incubated with *L. donovani* non-infected and infected macrophages. The supernatants of the medium were assayed to quantify pro-inflammatory cytokines (IL-12, IFN-γ and TNF-α), which are crucial for the successful treatment of VL and anti-inflammatory cytokines (IL-10 and IL-4), which participate in the exacerbation of the disease. The levels of cytokines were evaluated by ELISA. IL-12, IFN-γ and TNF-α all showed significant downregulation after infection (Fig. 5a–c). *Leishmania donovani* are found to be sensitive to DHCsA-d and SSG, thus the DHCsA-d and SSG treatments resulted in significant increases of IL-12, IFN-γ and TNF-α in *L. donovani* infected cells on the 24 h, 48 h and 72 h. However, the levels of these three cytokines did not change significantly in *L. donovani-*infected cells in the CsA-treated group compared to those in the untreated group.

The tendencies of variation of the cytokines IL-10 and IL-4 and NO and H_2_O_2_ production were identical. IL-10, IL-4, NO and H_2_O_2_ production showed significant enhancement after infection (Fig. 5d–g) but was observed to be significantly decreased in DHCSA-d and SSG- treated *L. donovani* at 24 h, 48 h and 72 h. However, the levels of IL-10, IL-4, NO and H_2_O_2_ did not change significantly in *L. donovani* infected cells in the CsA-treated group compared to those in the untreated group. Furthermore, the levels of all the five cytokines and NO and H_2_O_2_ production decreased over a period of time in all groups after treatment. The results of statistical tests were calculated (Table 2).

**Table 2:** Statistical tests for cytokines, NO and H_2_O_2_

		24 h	48 h	72 h
IL-12	N vs I-*L*. DD8	*t*_(12)_ = 2.4285 *P* = 0.0318	*t*_(12)_ = 2.9252 *P* = 0.0127	*t*_(12)_ = 2.87 *P* = 0.0141
	I-*L*. DD8 + CsA *vs* I-*L*. DD8	*t*_(12)_ = 0.8554 *P* = 0.4149	*t*_(12)_ = 0.9571 *P* = 0.3567	*t*_(12)_ = 1.4637 *P* = 0.1689
	I-*L*. DD8 + DHCsA-d *vs* I-*L*. DD8	*t*_(12)_ = 9.7045 *P* < 0.0001	*t*_(12)_ = 8.2368 *P* < 0.0001	*t*_(12)_ = 7.3545 *P* < 0.0001
	I-*L*. DD8 + SSG *vs* I-*L*. DD8	*t*_(12)_ = 9.6968 *P* < 0.0001	*t*_(12)_ = 8.7509 *P* < 0.0001	*t*_(12)_ = 7.4532 *P* < 0.0001
IFN-γ	N *vs* I-*L*. DD8	*t*_(12)_ = 2.3545 *P* = 0.0364	*t*_(12)_ = 3.5317 *P* = 0.0041	*t*_(12)_ = 4.4146 *P* = 0.0008
	I-*L*. DD8 + CsA *vs* I-*L*. DD8	*t*_(12)_ = 1.0839 *P* = 0.2997	*t*_(12)_ = 1.0695 *P* = 0.3059	*t*_(12)_ = 0.7226 *P* = 0.4838
	I-*L*. DD8 + DHCsA-d *vs* I-*L*. DD8	*t*_(12)_ = 23.9059 *P* < 0.0001	*t*_(12)_ = 22.2593 *P* < 0.0001	*t*_(12)_ = 19.4494 *P* < 0.0001
	I-*L*. DD8 + SSG *vs* I-*L*. DD8	*t*_(12)_ = 21.4421 *P* < 0.0001	*t*_(12)_ = 20.7228 *P* < 0.0001	*t*_(12)_ = 19.7626 *P* < 0.0001)
TNF-α	N *vs* I-*L*. DD8	*t*_(12)_ = 2.3822 *P* = 0.0346	*t*_(12)_ = 3.8117 *P* = 0.0025	*t*_(12)_ = 4.3674 *P* = 0.0009
	I-*L*. DD8 + CsA *vs* I-*L*. DD8	*t*_(12)_ = 2.0144 *P* = 0.0669	*t*_(12)_ = 2.1263 *P* = 0.0549	*t*_(12)_ = 1.9025 *P* = 0.0814
	I-*L*. DD8 + DHCsA-d *vs* I-*L*. DD8	*t*_(12)_ = 11.3431 *P* < 0.0001	*t*_(12)_ = 10.1318 *P* < 0.0001	*t*_(12)_ = 9.2065 *P* < 0.0001
	I-*L*. DD8 + SSG *vs* I-*L*. DD8	*t*_(12)_ = 12.1475 *P* < 0.0001	*t*_(12)_ = 10.9044 *P* < 0.0001	*t*_(12)_ = 9.4807 *P* < 0.0001
IL-10	N *vs* I-*L*. DD8	*t*_(12)_ = 17.7485 *P* < 0.0001	*t*_(12)_ = 17.021 *P* < 0.0001	*t*_(12)_ = 16.7054 *P* < 0.0001,
	I-*L*. DD8 + CsA *vs* I-*L*. DD8	*t*_(12)_ = 1.6069 *P* = 0.134	*t*_(12)_ = 1.6572 *P* = 0.1234	*t*_(12)_ = 0.8703 *P* = 0.4012
	I-*L*. DD8 + DHCsA-d *vs* I-*L*. DD8	*t*_(12)_ =11.2823 *P* < 0.0001	*t*_(12)_ = 11.3959 *P* < 0.0001	*t*_(12)_ = 12.8072 *P* < 0.0001
	I-*L*. DD8 + SSG vs I-*L*. DD8	*t*_(12)_ =11.9725 *P* < 0.0001	*t*_(12)_ = 12.1698 *P* < 0.0001	*t*_(12)_ = 13.2122 *P* < 0.0001
IL-4	N vs I-*L*. DD8	*t*_(12)_ = 11.8592 *P* < 0.0001	*t*_(12)_ = 11.2109 *P* < 0.0001	*t*_(12)_ = 9.6496 *P* < 0.0001
	I-*L*. DD8 + CsA *vs* I-*L*. DD8	*t*_(12)_ = 1.1525 *P* = 0.2715	*t*_(12)_ = 1.3592 *P* = 0.1991	*t*_(12)_ = 1.4827 *P* = 0.1639
	I-*L*. DD8 + DHCsA-d *vs* I-*L*. DD8	*t*_(12)_ = 6.8682 *P* < 0.0001	*t*_(12)_ = 6.4039 *P* < 0.0001	*t*_(12)_ = 5.8037 *P* < 0.0001
	I-*L*. DD8 + SSG *vs* I-*L*. DD8	*t*_(12)_ = 8.1166 *P* < 0.0001	*t*_(12)_ = 7.9485 *P* < 0.0001	*t*_(12)_ = 6.6847 *P* < 0.0001
NO	N *vs* I-*L*. DD8	*t*_(12)_ = 14.8865 *P* < 0.0001	*t*_(12)_ = 11.2109 *P* < 0.0001	*t*_(12)_ = 8.9723 *P* < 0.0001
	I-*L*. DD8 + CsA *vs* I-*L*. DD8	*t*_(12)_ = 0.6153 *P* = 0.5498	*t*_(12)_ = 0.6931 *P* = 0.5015	*t*_(12)_ = 0.5985 *P* = 0.5606
	I-*L*. DD8 + DHCsA-d *vs* I-*L*. DD8	*t*_(12)_ = 7.7965 *P* < 0.0001	*t*_(12)_ = 5.8653 *P* < 0.0001	*t*_(12)_ = 4.72 *P* = 0.0004
	I-*L*. DD8 + SSG vs I-*L*. DD8	*t*_(12)_ = 7.7822 *P* < 0.0001	*t*_(12)_ = 5.6942 *P* = 0.0001	*t*_(12)_ = 3.8402 *P* = 0.0024
H_2_O_2_	N *vs* I-*L*. DD8	*t*_(12)_ = 4.6596 *P* = 0.0006	*t*_(12)_ = 5.3572 *P* = 0.0002	*t*_(12)_ = 3.3676 *P* = 0.0056
	I-*L*. DD8 + CsA *vs* I-*L*. DD8	*t*_(12)_ = 0.9975 *P* = 0.3382	*t*_(12)_ = 1.222 *P* = 0.2452	*t*_(12)_ = 0.7519 *P* = 0.4666
	I-*L*. DD8 + DHCsA-d *vs* I-*L*. DD8	*t*_(12)_ = 4.6618 *P* = 0.0005	*t*_(12)_ = 5.405 *P* = 0.0002	*t*_(12)_ = 3.4004 *P* = 0.0053
	I-*L*. DD8 + SSG *vs* I-*L*. DD8	*t*_(12)_ = 4.6047 *P* = 0.0006	*t*_(12)_ = 5.2354 *P* = 0.0002	*t*_(12)_ = 3.0176 *P* = 0.0107

*Abbreviations*: N, non-infected macrophages; I-*L*. DD8, macrophages infected with *L*. DD8; I-*L*. DD8 + SSG, SSG adding after macrophages infected with *L*. DD8; I-*L*. DD8 + CsA, CsA adding after macrophages infected with *L*. DD8; I-*L*. DD8 + DHCsA-d, DHCsA-d adding after macrophages infected with *L*. DD8

### Preparation of *Ld*CyPA antibody

**Fig. 6:**
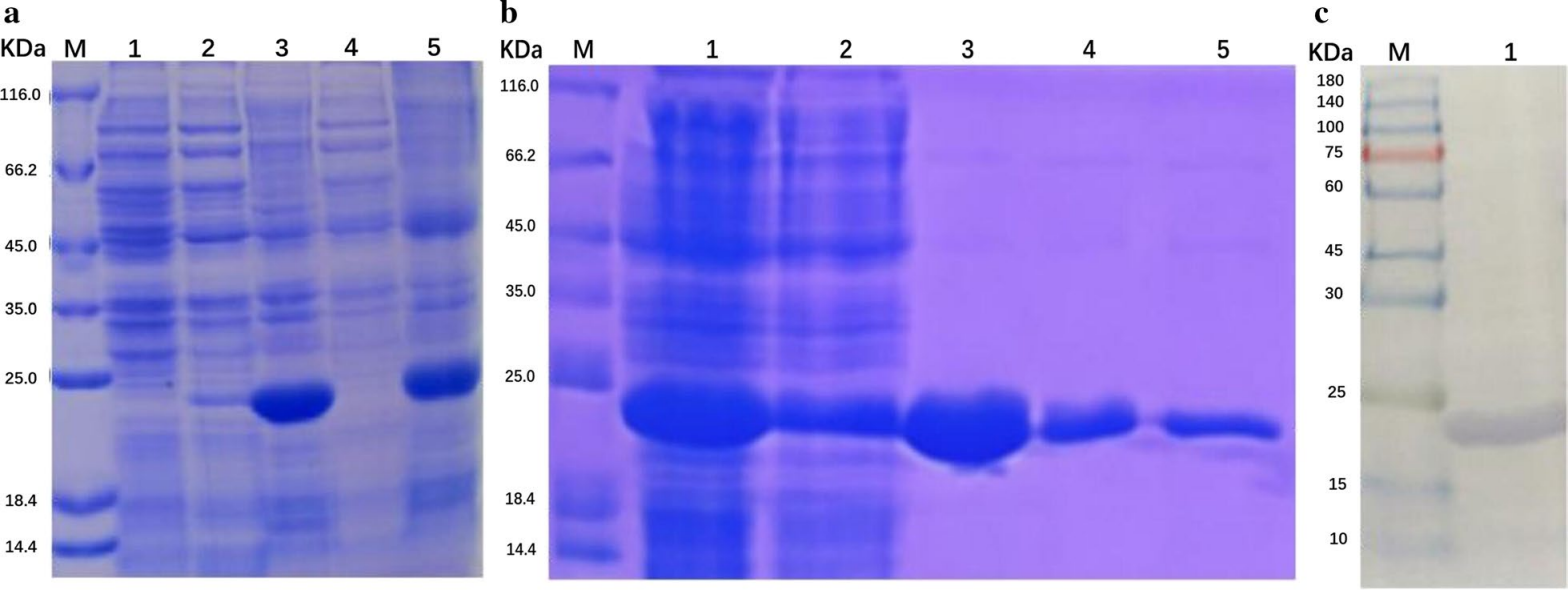
Cloning and expression of *Ld*CyPA protein. **a** SDS-PAGE analysis of fusion protein in *Escherichia coli* BL21 (containing pET28a). Lane M: protein marker; Lane 1: total protein before induction; Lane 2: 20 °C supernatant; Lane 3: 20 °C precipitate; Lane 4: 37 °C supernatant; Lane 5: 37 °C precipitate (target fusion protein). **b** SDS-PAGE analysis of eluted protein. Lane M: protein marker; Lane 1: sample solution; Lane 2: penetrating fluid; Lane 3: 20 mM Imidazole-eluted protein; Lane 4: 50 mM Imidazole-eluted protein; Lane 5: 500 mM Imidazole-eluted protein (target *Ld*CyPA protein). **c** Western blotting-based identification of the purified *Ld*CyPA protein. Lane M: protein markers; Lane 1: purified *Ld*CyPA protein

The *Ld*CyPA gene was successfully amplified by PCR and ligated into the plasmid pET-28a. The construction of the recombinant plasmid was verified by double enzyme digestion. The recombinant pET-28a vector was then transferred into BL21 cells to express the fusion protein, which was purified using Ni-NTA agarose affinity chromatography and verified by the sodium dodecyl sulfate polyacrylamide gel electrophoresis (Fig. 6a, b). Further, western blotting demonstrated that a purified *Ld*CyPA protein was obtained (Fig. 6c). The concentration of the *Ld*CyPA protein was 3.87 mg/ml, as estimated by the BCA method. Serum samples of rabbits immunized with *Ld*CyPA were collected for detection of the titers on 25 and 42 days. Since the titers were greater than 1:50,000 according to ELISA, the sera of these rabbits were further purified to obtain specific *Ld*CyPA antibodies.

### The expression of CyPA and *Ld*CyPA after drug inhibition

**Fig. 7:**
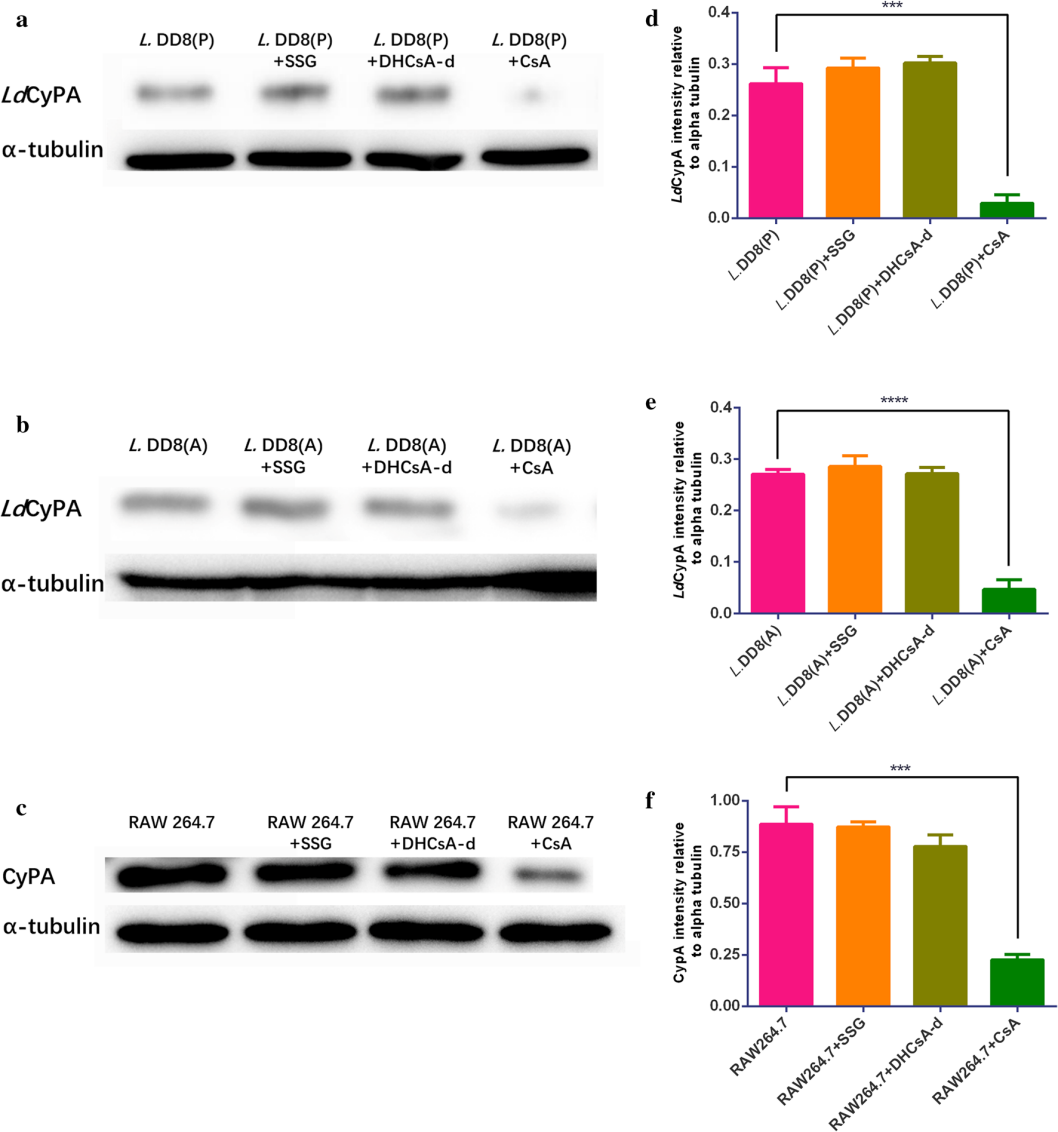
*Ld*CyPA and CyPA expression after treatment with three drugs. **a** *Ld*CyPA expression levels of promastigotes treated with SSG, DHCsA-d, or CsA for 48 h; *Ld*CyPA polyclonal antibody and α-tubulin antibody were used as primary antibodies. **b** *Ld*CyPA expression levels of intracellular amastigotes treated with SSG, DHCsA-d, or CsA for 48 h; *Ld*CyPA polyclonal antibody and α-tubulin antibody used as primary antibodies. **c** CyPA expression levels of RAW 264.7 mouse macrophages co-cultured with SSG, DHCsA-d, or CsA at 48 h; CyPA and α-tubulin antibodies used as primary antibodies. Densitometry plot of *Ld*CyPA expression in promastigotes (**d**) and amastigotes (**e**) normalized by an endogenous control, α-tubulin. **f** Densitometry plot of CyPA expression in RAW264.7 cells, normalized by an endogenous control, α-tubulin. Data are represented as the mean ± SD and represent the values of one of the 3 independent experiments. ***P < 0.001, ****P < 0.0001. *Abbreviations*: A, intracellular amastigote; P, promastigote

The expression of *Ld*CyPA in promastigotes (*t*_(4)_ = 11.45, *P* = 0.0003) and intracellular amastigotes (*t*_(4)_ = 18.85, *P* < 0.0001) treated with CsA and the expression of CyPA in RAW 264.7 cells (*t*_(4)_ = 12.95, *P* = 0.0002) treated with CsA were found to be significantly downregulated compared to those in the untreated cells (Fig. 7a–f). However, no significant changes were found in the expression levels of *Ld*CyPA in promastigotes (*t*_(4)_ = 2.076, *P* = 0.1065; *t*_(4)_ = 1.45, *P* = 0.2206) and intracellular amastigotes (*t*_(4)_ = 0.1112, *P* = 0.9168; *t*_(4)_ = 1.131, *P* = 0.3213) and CyPA in RAW 264.7 cells (*t*_(4)_ = 1.862, *P* = 0.1361; *t*_(4)_ = 0.2635, *P* = 0.8052) treated with DHCsA-d or SSG compared to those in the untreated cells (Fig. 7a–f). Densitometric analysis results showed lower *Ld*CyPA levels in promastigotes and amastigotes treated with CsA than in those treated with DHCsA-d or SSG (Fig. 7d, e) and lower CyPA levels were seen in RAW 264.7 cell co-cultured with CsA than those co-cultured with DHCsA-d or SSG (Fig. 7f).

### Dihydrocyclosporin A induced high cytotoxicity in RAW264.7 cells

The safety profile of CsA, DHCsA-d, or SSG was examined using a mammalian cytotoxicity test using RAW 264.7 cells. CsA and DHCsA-d demonstrated cytotoxicity towards macrophages, showing 50% cytotoxic concentration (CC_50_) values of 15.91 μM (CsA, 24 h), 12.65 μM (CsA, 48 h), 7.98 μM (DHCsA-d, 24 h) and 6.65 μM (DHCsA-d, 48 h); by contrast, SSG exhibited a CC_50_ value > 120 μM (Table 3).

**Table 3:** CC_50_ of CsA, DHCsA-d, or SSG used for treatment of RAW 264.7 cells

Compound	Cytotoxicity (RAW 264.7) CC_50_	
	24 h	48 h
	Mean ± SD (µM)	Mean ± SD (µM)
CsA	15.91 ± 0.2	12.65 ± 1.2
DHCsA-d	7.98 ± 0.4	6.65 ± 0.7
SSG	> 120	> 120

*Note*: Data are represented as the mean ± SD of three independent experiments

*Abbreviations*: CC_50_, 50% cytotoxic concentration; SD, standard deviation

## Discussion

VL is potentially fatal to patients if left untreated. Because it causes severe immunosuppression, it makes patients more susceptible to secondary microbial infections [25]. Due to the drug resistance, high cost, toxic side effects, and lengthy treatment regimens of pentavalent antimonials, it is necessary to find an alternative therapeutic drug for VL. As mentioned above, CsA and its non-immunosuppressive derivatives were considered to be potential therapeutic drugs to treat VL. However, whether CsA and its derivatives can be used as therapeutic drugs for VL has been controversial for many years.

In this study, the inhibitory effects of CsA and its derivative DHCsA-d on promastigotes and intracellular amastigotes of *L. donovani* were assessed. The number of intracellular amastigotes decreased significantly compared to that in the untreated group after administration of DHCsA-d or SSG. However, the number of intracellular amastigotes distinctly increased after the administration of CsA. These results indicated that CsA and DHCsA-d had anti-leishmanial effects on promastigotes, while CsA was found to promote the infection of intracellular amastigotes.

The role of CsA in promoting intracellular amastigote infection was confirmed by assessing morphological observation and cytokine expression levels in this study. According to ultrastructure observations, after treatment with DHCsA-d or SSG, there were obvious changes in the mitochondria of promastigotes and amastigotes. The mitochondria were completely disorganized; this result was similar to the findings regarding ultrastructural changes in the mitochondria of *L. amazonensis* treated with itraconazole and ravuconazole [26, 27]. However, no obvious changes in mitochondria were observed after treatment with CsA.

Appropriate activation of macrophages, which are the major effector cells responsible for elimination of leishmania, is crucial for eliminating intracellular *Leishmania* [28]. Therefore, the effects of three pro-inflammatory cytokines (IL-12, IFN-γ and TNF-α), which are crucial for the successful treatment of VL and anti-inflammatory cytokines (IL-4 and IL-10), which participate in the exacerbation of the disease, secreted by macrophages, were evaluated by ELISA in this study. We found that the levels of IL-4 and IL-10 were significantly increased, while those of IL-12, IFN-γ and TNF-α were significantly decreased after phagocytosis of *L. donovani* by macrophages. These results are supported by the cytokine expression in VL patients who were infected with *L. donovani* [29]. High levels of IL-10 were found in the serum of these VL patients, which could inhibit the activity of pro-inflammatory cytokines, such as IFN-γ and TNF-α [29]. Macrophages produce IL-12 to promote a Th1 response during leishmanial infection, which protects the host and controls parasite replication [30, 31]. Meanwhile, TNF-α and IFN-γ, implicated to be involved in the activation of macrophages, were also observed to play a role in preventing parasite replication [32]. Previous studies also verified that drugs which have inhibitory effects on *L. donovani*, could decrease IL-10 and IL-4 in VL mice [4, 33, 34]. In this study, the reduction of parasite burden was supported by the increase of IL-12, IFN-γ and TNF-α and decrease of IL-4 and IL-10 after DHCsA-d or SSG treatments. On the contrary, the levels of IL-12, IFN-γ and TNF-α decreased and IL-4 and IL-10 increased after CsA treatment compared with those of the untreated group.

In the first few hours of leishmanial infection within macrophages, the most effective anti-parasitic response of the macrophages is the production of reactive oxygen species (ROS) and reactive nitrogen species (RNS) [35, 36]. The two major reactive species in macrophages are superoxide (O_2_^−^) and nitric oxide (NO) [37, 38]. It is observed that if drugs have ideal anti-parasitic effects, the NO and H_2_O_2_ productions of leishmania-infected macrophages decrease [4, 39]. In our research, elevated levels of NO and H_2_O_2_ were found in leishmania-infected macrophages and a strong inhibition of NO and H_2_O_2_ metabolites was observed upon treatment with DHCsA-d or SSG. However, an increase of these metabolites was observed after treatment with CsA. Meissner et al. [40] also found that CsA did not rely on reactive nitrogen to kill *L. major* by macrophages and the NO production was also not increased after CsA treatment.

Cyclophilin A (CyPA), which possesses a peptidylprolyl isomerase (PPIase) activity, is an important member of a highly conserved family of human multi-functional proteins called cyclophilins (CyPs) [16, 17]. CyPA, having an immunosuppressive action, is characterized by the binding of cyclic peptide inhibitor, CsA, which inhibits protein phosphatase calcineurin and T cell-mediated immunity [11]. Parasite cyclophilins are also known to be potential drug targets, because the immunosuppressive drug CsA can be potentially used as an antiparasitic drug against *Leishmania tropica* [7], *Schistosoma mansoni* [22] and *Plasmodium falciparum* [41]. *Trypanosoma cruzi* cyclophilin (*Tc*CyP19) is suggested to play a protective role in the survival of the infected tissue and allow parasite persistence [20]. The *Leishmania* cyclophilin of 19 kDa, *Ld*CyPA, has a 79–81% protein sequence identity with the *Tc*CyP19 [42]. In this study, western blotting showed a decreased expression of CyPA and *Ld*CyPA after CsA treatment, while that of DHCsA-d or SSG groups showed no significant changes. These results confirmed observations in a previous study demonstrating that cell CyPA can inhibit the immunity of macrophages by binding to CsA and that *Ld*CyPA can promote the intracellular viability and proliferation of leishmania by binding to CsA. According to the above results, since CsA is not shown to have an inhibitory effect on intracellular amastigotes, we believe that CsA should not be recommended for VL treatment.

DHCsA-d had an excellent inhibitory effect on both promastigotes and intracellular amastigotes and did not inhibit the immunological effects of macrophages or promote leishmania infection. Unfortunately, the CC_50_ of DHCsA-d was only 7.98 μM (24 h) and 6.65 μM (48 h) in this study, suggesting its high cytotoxicity. Therefore, DHCsA-d is also not recommended for VL treatment. This study demonstrated that CsA and DHCsA-d might not be ideal antileishmanial agents due to various limitations, which may provide a reference for subsequent researches.

## Conclusions

The results of our study have resolved the dispute regarding the efficacy of CsA and DHCsA-d for VL treatment. CsA did not show a significant inhibitory effect on the intracellular amastigotes of *L. donovani*. DHCsA-d significantly inhibited promastigotes and intracellular amastigotes, but it was found to be highly cytotoxic. We believe that CsA and DHCsA-d should not be recommended as antileishmanial drugs. Research towards new therapeutic drugs for VL has a long way to go and further studies are required to develop appropriate anti-leishmanial drugs.

## Data Availability

The datasets supporting the conclusions of this article are included within the article.

## Abbreviations

CsA: Cyclosporine A
DHCsA-d: Dihydrocyclosporin A
SSG: Sodium stibogluconate
CyPA: Cyclophilin A
*Ld*CyPA: *Leishmania donovani* Cyclophilin A
VL: Visceral leishmaniasis
Sb^V^: Pentavalent antimonials
IC_50_: 50% inhibitory concentration
CC_50_: 50% cytotoxic concentration
ELISA: Enzyme-linked immunosorbent assay
